# Supplementary figures and images for: HIV-1 infection does not confer intrinsic resistance to cell death induced by cytotoxic T lymphocytes
Source: bioRxiv. 2026 Apr 30:2026.03.23.713717. Originally published 2026 Mar 25. Preprint. [Version 2] doi: 10.64898/2026.03.23.713717 (PMC13041980; doi:10.64898/2026.03.23.713717)

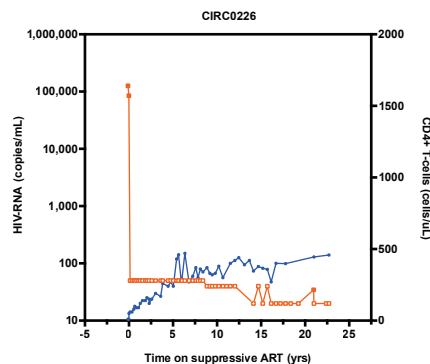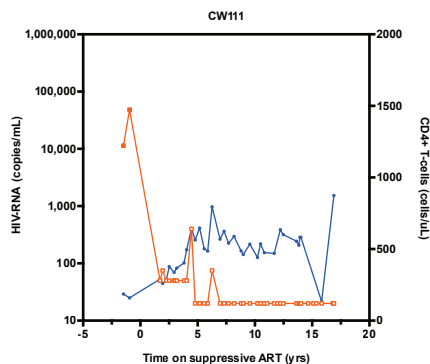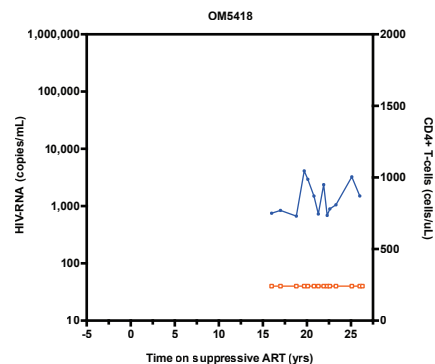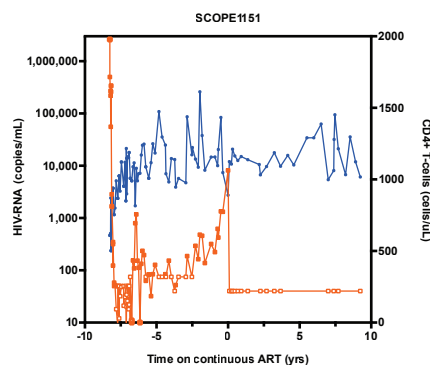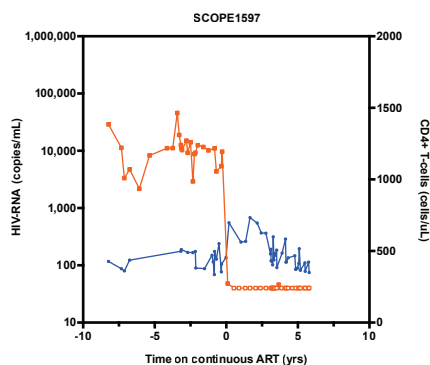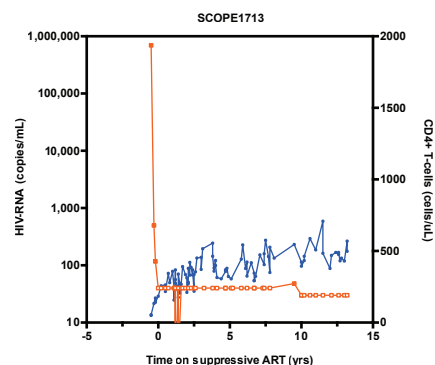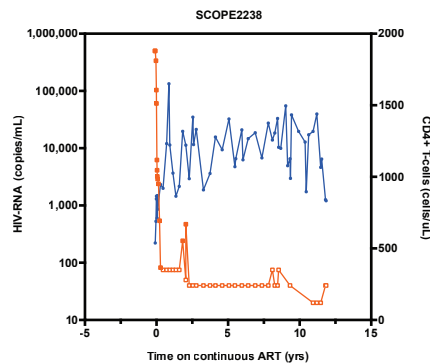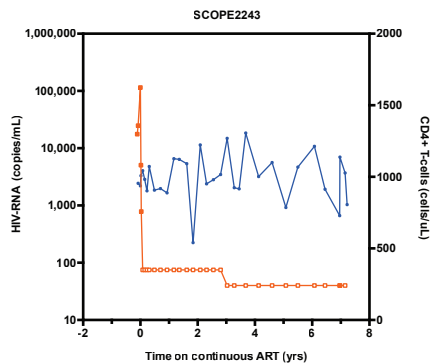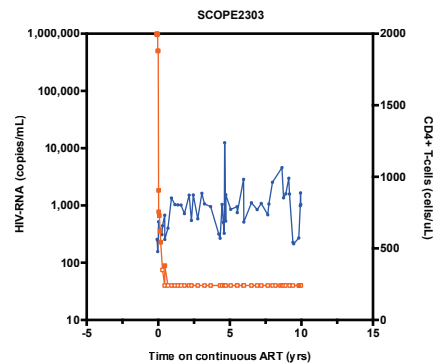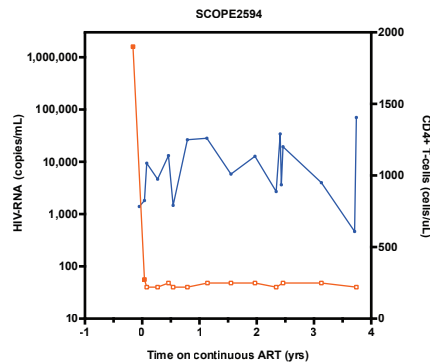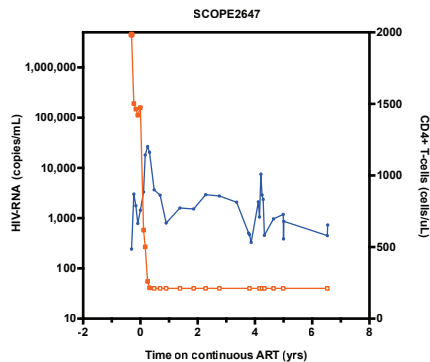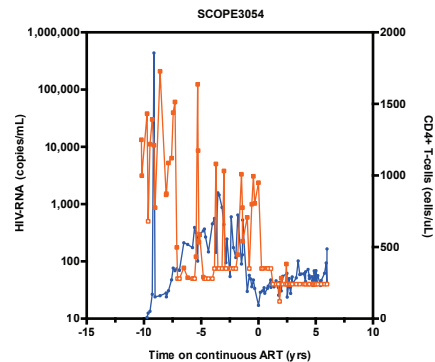

Supplement: Supplement 1 [file media-1.pdf]
